# The MC4R genetic variants are associated with lower visceral fat accumulation and higher postprandial relative increase in carbohydrate utilization in humans

**DOI:** 10.1007/s00394-019-01955-0

**Published:** 2019-04-03

**Authors:** Edyta Adamska-Patruno, Joanna Goscik, Przemyslaw Czajkowski, Katarzyna Maliszewska, Michał Ciborowski, Anna Golonko, Natalia Wawrusiewicz-Kurylonek, Anna Citko, Magdalena Waszczeniuk, Adam Kretowski, Maria Gorska

**Affiliations:** 1 Clinical Research Centre, Medical University of Bialystok, M.C. Sklodowskiej-Curie 24A, 15-276 Bialystok, Poland; 2 Centre for Experimental Medicine, Medical University of Bialystok, M.C. Sklodowskiej-Curie 24A, 15-276 Bialystok, Poland; 3 Department of Endocrinology, Diabetology and Internal Medicine, Medical University of Bialystok, M.C. Sklodowskiej-Curie 24A, 15-276 Bialystok, Poland; 4 Department of Dietetics and Clinical Nutrition, Medical University of Bialystok, Mieszka I-go 4B, 15-054 Bialystok, Poland

**Keywords:** Melanocortin-4-receptor MC4R gene, Nutrigenetic, Postprandial carbohydrate utilization, Body fat accumulation, Visceral fat content, Obesity

## Abstract

**Purpose:**

The interactions between lifestyle and genetic factors play an important role in obesity development. Mutations in melanocortin-4-receptor (MC4R) gene are one of the most common cause of monogenic obesity, however, the functional effects of polymorphic variants near MC4R gene in general populations remain uncertain. The aim of our study was to analyze whether the common single nucleotide polymorphisms (SNPs) of MC4R gene influence the food preferences, physical activity, body fat content and distribution, as well as fasting and postprandial energy expenditure and substrates utilization.

**Methods:**

We genotyped previously identified MC4R SNPs: rs17782313, rs633265, rs1350341, rs12970134 in 927 subjects, who underwent anthropometric, total body fat content, visceral (VAT) and subcutaneous adipose tissue (SAT) measurements, and daily physical activity and dietary intake analysis. In randomly selected 47 subjects the energy expenditure, carbohydrate and lipid utilizations were evaluated in fasting state and after high-carbohydrate and control meals intake.

**Results:**

We found the significant associations between studied SNPs of MC4R gene and VAT and VAT/SAT ratio. Moreover, the GG genotype carriers of rs1350341, who had the lowest VAT accumulation (*p* = 0.012), presented higher relative increase in postprandial carbohydrate utilization (*p* = 0.013, *p* = 0.024).

**Conclusions:**

We have observed that common SNPs of the MC4R gene influence the body fat content and distribution, as well as relative increase in postprandial carbohydrate utilization. We believe that our study may help to understand better the impact of MC4R gene on obesity development, and to help to provide personalized prevention/treatment strategies to fight against obesity and its metabolic consequences.

## Introduction

The prevalence of obesity is rapidly increasing worldwide, and it has become an important clinical problem [1]. This multifactorial disorder is a major risk factor of type 2 diabetes mellitus (T2DM), hypertension, hyperlipidemia, cardiovascular diseases and the increase in the obesity development can lead to further morbidity and mortality [2, 3]. The large-scale genome-wide association studies (GWAS) and meta-analyzes have revealed over 52 new loci, including single nucleotide polymorphisms (SNPs) in or near the fat mass and obesity-associated (FTO) gene, melanocortin-4 receptor (MC4R) gene, Peroxisome proliferator-activated receptor gamma (PPAR), brain-derived neurotrophic factor (BDNF), neuronal growth regulator 1 (NEGR1), BTB/POZ domain-containing protein (KCTD15), and many other genes associated with BMI, waist circumference and/or waist–hip ratio (WHR) [4]. Some of the SNPs may be associated with the weight gain due to larger amounts of consumed food, due to deprivation of postprandial satiety, as well as due to deprivation of energy expenditure and/or substrate utilization [5–10]. In our previous studies, we have noticed that some of the metabolic changes may appear only in postprandial state, and that postprandial metabolism can be dependent on the meal content [11, 12], as well as on the carried genetic variants [13, 14]. It is beyond any doubts that genetic factors predispose to obesity, however, obesity should not be considered only as a genetic disorder. The increasing prevalence of obesity and T2DM in the modern environment generally can be contributed by the excessive energy intake, and/or by changes in dietary habits dependent on the age, as well as by diminished physical activity, but also it is already known, that these diseases may be influenced by interaction between lifestyle and genetic factors [6, 15, 16], which can modulate the impact of the environment on each individual’s risk.

The MC4R gene mutations are one of the most common form of monogenic obesity [4, 17, 18], however the functional effects of polymorphic variants in general population remain uncertain. The MC4Rs are expressed in several sites in the brain, including hypothalamus, forebrain and hindbrain, which had been implicated in central energy balance regulation. The activation of MC4Rs, as a part of melanocortin system, increases energy expenditure, insulin sensitivity and it has been implicated in the food intake regulation. Rich in MC4Rs are also amygdala and latheral hypothalamus, the important centers of taste perception, which connect hypothalamus with mesolimbic reward system. Moreover, melanocortin system signaling in the latheral hypothalamus has been shown to affect the response to a high-fat diet [19]. Some of the rare MC4R gene mutations are associated with binge eating, excessive hunger, meal choices, food-seeking behaviors and hyperphagia, as well as with influencing the energy expenditure [19–22]. The GWAS have identified also some common genetic variants near the MC4R gene (rs17782313, rs17700633), which are related to the increased fat mass content, body weight, obesity [17] and T2DM [23], but it is still unclear whether dietary factors may influence the relation between some genetic variants and obesity. Only few studies have been attempted so far and results are conflicting. Some authors suggest that SNPs 12970134 influences energy and dietary fat intake [24, 25], as well as eating behaviors [26], while other authors suggest that rs12970134 nor rs17782313 near the MC4R gene do not influence food intake, nor preferences for specific food items [21]. It is already known that interactions between MC4R gene with diet play a significant role in obesity and T2DM development [27], but the mechanisms by which different variants of SNPs near MC4R gene may influence the metabolic changes that lead to obesity, have not been known particularly yet. Therefore an early assessment and detection of any possible dependencies between the MC4R risk alleles and diet, and next the modifications of their dietary patterns based on these results, can be an efficient strategy to prevent obesity and its metabolic consequences. To our knowledge, associations between SNPs near MC4R gene and possible metabolic expressions, such as energy expenditure, have been investigated only by Kring et al. [28], and only for rs17782313, but substrates utilization after meal intake, dependently on various SNPs near MC4R gene, have not been studied yet.

The aim of our study was to analyze whether some of the common genetic variants near MC4R gene, which have associated with obesity in GWAS, may influence the body fat content, body fat distribution, visceral fat accumulation, as well as dietary intake, physical activity, fasting and postprandial energy expenditure and substrates utilization, after meals with different macronutrient content. This trial was registered at www.clinicaltrials.gov as NCT03792685.

## Materials and methods

### Participants

We genotyped previously identified MC4R SNPs: rs17782313, rs633265, rs1350341, rs12970134 in 927 subjects (the mean age 40.22 ± 0.47 years, mean BMI 28.17 ± 0.22 kg/m^2^, 473 men and 454 women). The 597 of individuals were overweight/obese, with BMI ≥ 25 kg/m^2^ (the mean age 44.20 ± 0.58 years; mean BMI 31.37  0.24 kg/m^2^), and 330 were healthy volunteers with normal weight, BMI < 25 kg/m^2^ (mean age 33.08 ± 0.64 years, mean BMI 22.39 ± 0.11 kg/m^2^). Participants were sent by primary care physicians as an apparently healthy people, and were recruited for a cohort study, described previously [14, 29]. The clinical characteristics of the studied population, determined by investigated genotypes, are reported in Tables 1, 2, 3, and 4.

**Table 1:** The anthropometric and body composition characteristics of rs17782313 genotypes

rs17782313	C/C	C/T	T/T	*p* value
*N* (women/men)	27 (10/17)	316 (145/171)	584 (299/285)	
Genotype frequency	0.029	0.341	0.630	> 0.05
BMI (kg/m^2^)	30.05 ± 1.27	28.47 ± 0.37	27.90 ± 0.27	0.14
WHR	0.94 ± 0.02	0.93 ± 0.01	0.92 ± 0.00	0.15
Fat mass (kg)	29.94 ± 2.51	25.98 ± 0.82	25.76 ± 0.57	0.32
Fat mass (%)	32.52 ± 1.53	29.72 ± 0.62	30.31 ± 0.43	0.36
Visceral fat (cm^3^)	166.61 ± 30.02	105.10 ± 5.90	94.04 ± 3.24	< 0.001
Visceral fat (%)	42.28 ± 3.56	37.09 ± 0.89	35.63 ± 0.49	0.024
Subcutaneous fat (cm^3^)	200.89 ± 22.34	156.43 ± 5.28	160.16 ± 4.07	0.09
Subcutaneous fat (%)	57.72 ± 3.56	62.91 ± 0.90	64.28 ± 0.51	0.033
Visceral/subcutaneous fat ratio	0.86 ± 0.12	0.69 ± 0.04	0.60 ± 0.02	0.001

**Table 2:** The anthropometric and body composition characteristics of rs633265 genotypes

rs633265	G/G	G/T	T/T	*p* value
*N* (women/men)	317 (161/156)	472 (233/239)	138 (60/78)	
Genotype frequency	0.342	0.509	0.149	> 0.05
BMI (kg/m^2^)	27.63 ± 0.35	28.36 ± 0.31	28.43 ± 0.52	0.25
WHR	0.92 ± 0.01	0.92 ± 0.00	0.94 ± 0.01	0.22
Fat mass (kg)	25.13 ± 0.72	26.27 ± 0.69	26.30 ± 1.11	0.49
Fat mass (%)	29.92 ± 0.57	30.34 ± 0.51	29.93 ± 0.80	0.83
Visceral fat (cm^3^)	93.14 ± 4.27	96.95 ± 4.22	120.98 ± 9.58	0.007
Visceral fat (%)	35.40 ± 0.67	36.20 ± 064	38.48 ± 1.33	0.07
Subcutaneous fat (cm^3^)	160.70 ± 5.38	155.26 ± 4.43	172.35 ± 8.78	0.18
Subcutaneous fat (%)	64.60 ± 0.67	63.66 ± 0.67	61.61 ± 1.32	0.097
Visceral/subcutaneous fat ratio	0.60 ± 0.02	0.64 ± 0.02	0.72 ± 0.05	0.05

**Table 3:** The anthropometric and body composition characteristics of rs12970134 genotypes

rs12970134	A/A	A/G	G/G	*p* value
*N* (women/men)	41 (18/23)	363 (172/191)	523 (265/258)	
Genotype frequency	0.044	0.392	0.564	> 0.05
BMI (kg/m^2^)	30.63 ± 1.10	28.32 ± 0.34	27.88 ± 029	0.03
WHR	0.95 ± 0.02	0.93 ± 0.00	0.92 ± 0.00	0.12
Fat mass (kg)	31.58 ± 2.21	25.78 ± 0.73	25.72 ± 0.62	0.03
Fat mass (%)	33.90 ± 1.31	29.86 ± 0.56	30.20 ± 0.47	0.07
Visceral fat (cm^3^)	152.13 ± 25.21	101.52 ± 4.93	95.29 ± 3.63	0.002
Visceral fat (%)	38.29 ± 3.36	36.90 ± 0.76	35.73 ± 0.53	0.31
Subcutaneous fat (cm^3^)	213.25 ± 16.35	154.61 ± 4.66	161.10 ± 4.48	0.003
Subcutaneous fat (%)	61.72 ± 3.36	63.10 ± 0.76	64.17 ± 0.56	0.37
Visceral/subcutaneous fat ratio	0.76 ± 0.12	0.68 ± 0.03	0.60 ± 0.02	0.02

**Table 4:** The anthropometric, body composition, daily physical activity, energy and macronutrients intake characteristics of rs1350341 genotypes

rs1350341	A/A	A/G	G/G	*p* value
*N* (women/men)	138 (62/76)	457 (232/225)	304 (160/144)	
Genotype frequency	0.154	0.508	0.338	> 0.05
BMI (kg/m^2^)	28.54 ± 0.53	28.49 ± 0.32	27.76 ± 0.36	0.28
WHR	0.93 ± 0.01	0.93 ± 0.00	0.92 ± 0.01	0.35
Fat mass (kg)	26.64 ± 1.11	26.66 ± 0.71	25.62 ± 0.74	0.58
Fat mass (%)	30.35 ± 0.79	30.69 ± 0.52	30.45 ± 0.58	0.99
Visceral fat (cm^3^)	122.28 ± 9.70	99.50 ± 4.39	95.26 ± 4.47	0.012
Visceral fat (%)	38.55 ± 1.35	36.41 ± 0.66	35.36 ± 0.69	0.07
Subcutaneous fat (cm^3^)	174.06 ± 8.82	158.01 ± 4.57	164.45 ± 5.57	0.22
Subcutaneous fat (%)	61.54 ± 1.34	63.45 ± 0.69	64.64 ± 0.69	0.09
Visceral/subcutaneous fat ratio	0.72 ± 0.05	0.65 ± 0.03	0.60 ± 0.02	0.046
Energy intake (kcal/day)	1758.72 ± 73.86	1813.25 ± 43.21	1901.84 ± 58.94	0.27
Protein intake (g/day)	80.19 ± 3.13	83.22 ± 1.87	87.95 ± 2.58	0.13
Protein intake (% of energy)	18.8 ± 0.01	18.9 ± 0.00	19.2 ± 0.00	0.75
Fat intake (g/day)	61.65 ± 3.32	62.86 ± 2.52	68.26 ± 2.72	0.26
Fat intake (% of energy)	30.6 ± 0.01	30.2 ± 0.00	31.7 ± 0.01	0.13
Carbohydrate intake (g/day)	227.58 ± 9.59	231.04 ± 5.19	242.31 ± 8.00	0.36
Carbohydrate intake (% of energy)	48.1 ± 0.01	47.8 ± 0.01	47.4 ± 0.01	0.75
Physical activity (MET)	9971 ± 635	9653 ± 377	8939 ± 486	0.35

### Anthropometric measurements

The weight and height were measured by the trained researchers in a standardized way [30]. All individuals underwent body weight and body composition analysis: body fat content (bioelectrical impedance analysis, InBody 220, Biospace, Korea), visceral abdominal adipose tissue (VAT) and subcutaneous abdominal adipose tissue (SAT) by a bioelectrical impedance analysis (Maltron 920-2 BioScan, Maltron International Ltd, UK). Body Mass Index (BMI) was calculated as body weight in kilograms divided by the square of height in meters. A waist circumference was measured midway between the iliac crest and the lower costal margin and hip circumference was measured at the maximum protrusion of the gluteal region. Waist-Hip Ratio was calculated as the waist circumference in centimeters divided by the hip circumference in centimeters.

### The physical activity and dietary intake analyses

In all of the volunteers the daily physical activity was estimated by an International Physical Activity Questionnaire-Long Form (IPAQ-LF), which is a self-administered questionnaire and it has acceptable validity to assess the levels of physical activity [31]. The results were expressed as MET (metabolic equivalent)-min per week (MET level × minutes of activity × events per week). The 3-day food diaries analyses were performed in a group of 623 subjects, as previously described [32, 33]. Portions of food were estimated by comparing with color photographs for each portion size (albums), as well as subjects were asked to weight food if possible. The daily energy, carbohydrates, fats and proteins intake were analyzed using Dieta 4 software.

### The pre- and post-meal tests

In the randomly selected 47 healthy subjects the standardized meal tests were performed, accordingly to previously described procedures [11–13, 34]. Taking into consideration the fact that investigated factors may be characterized by sexual dimorphism [35], only male participants were included in the study group. All subjects were free from the prediabetes states, T2DM, endocrine, renal, hepatic and gastrointestinal disorders, as well as without any treatments that might affect the results. A group of 47 men was chosen in order to fulfill inclusion criteria. Since the overall sample size was bigger that 47, a 47-combination without repetition from 350 was used to select the desired sample (350 men fulfilled the conditions). The subjects were instructed to avoid coffee, alcohol and excessive physical exercise on the day before each test, and to maintain their regular lifestyle throughout the study. Briefly, after an overnight fast, the subjects arrived at the laboratory and were positioned in bed in the quiet room with thermoneutral conditions (22–25 °C) for at least 30 min of rest. Resting energy expenditure and substrates utilization were determined by a computed open-circuit indirect calorimetry- the noninvasive method recommended to measure REE [36], based on resting oxygen uptake and resting carbon dioxide production, by a ventilated canopy Vmax Encore 29n System (Viasys HealthCare, Yorba Linda, CA, USA), which is one of the most valid gas analysis system [37]. Next, subjects received a standardized high-carbohydrate (HC) meal (300 ml, Nutridrink Fat Free, Nutricia Poland, Warsaw, Poland), which provided 450 kcal (89.3% of energy from carbohydrate, 10.7% of energy from protein, and 0% of energy from fat). Moreover, after 1–2 weeks, the 24 individuals who agreed to participate the second meal challenge test, received an isocaloric (450 kcal) control (C) meal (360 ml, Cubitan, Nutricia Poland, Warsaw, Poland), which provided 45.1% of energy from carbohydrate, 29.7% of energy from protein, and 25.2% of energy from fat. Subjects were asked to consume the entire meal within 10 min. During meal test only 150 ml of water was allowed to consume. Fasting (0 min) and postprandial (60, 120, 180, 240 min) energy expenditure, carbohydrate and fat utilization were evaluated for a further 4h after meal intake. The measurements were performed for 30 min and were expressed as kcal or mg/min/ffm (fat free mass).

### Genetic analyses

DNA was extracted from the peripheral blood leukocytes using a classical salting out method. All SNPs were genotyped by TaqMan SNP technology from ready to use human assays library (Applied Biosystems, USA) using a high throughput genotyping system—OpenArray from Life Technologies (USA). SNPs analysis was performed in duplicate following the manufacturer’s instructions. As a negative control, we used a sample without template. The negative control was helpful for measuring any false positive signal caused by contamination. No significant deviation from Hardy Weinberg equilibrium was observed for either of the SNPs in this study (all *p* > 0.05).

### Statistical analysis

In order to check whether the genotypes’ frequencies did not differ significantly (in the statistical manner), i.e. reflected the populational distribution, a series of proportions’ tests were conducted. Statistically significant differences between groups determined by genotypes were estimated either by the Kruskal–Wallis test or with the use of one-way ANOVA depending on fulfilling the following assumptions: homogeneity of variances, approximately normal distribution within groups. One-way ANOVA was carried out when both conditions were accomplished. Selected quantitative features, that constituted the subject of the study, were considered as dependent variables. To apprise which genotypes caused the particular test to be significant at a significance level 0.05, post-hoc analysis was performed applying either the Wilcoxon rank-sum test or the t-test (for all pairwise comparisons). Assumption of homogeneity of variances was checked with the Levene test, while the normality was verified with the Shapiro–Wilk test. Due to the problem of multiple testing false discovery rate (FDR) *p* value adjustment was used [38]. For all calculations the R software environment [39] was employed.

## Results

The anthropometric and body composition characteristics of studied group by genotypes are presented in Tables 1, 2, 3, and 4. The genotype frequencies were compared with those expected under Hardy–Weinberg equilibrium (*p* < 0.05). The characteristic of the meal-test substudy population is presented in Table 5. The genotypes frequencies in the meal-test substudy population are presented in Table 6. The selected group reflects the population (in terms of genotypes’ distribution), since none of the test gave a statistically significant result (Table 7).

**Table 5:** The characteristic of the meal-test substudy population

rs12970134	A/A	A/G	G/G	*p* value
Age (years)	36.02 ± 0	36.32 ± 1.95	38.12 ± 2.26	0.55
BMI (kg/m^2^)	28.25 ± 0	27.32 ± 1.17	30.30 ± 1.62	0.1
Fat mass (%)	30.00 ± 0	21.45 ± 1.56	28.18 ± 1.77	0.01

rs17782313	C/C	C/T	T/T	*p* value
Age (years)	NA	35.38 ± 2.07	28.45 ± 2.00	0.29
BMI (kg/m^2^)	NA	27.75 ± 1.33	29.64 ± 1.43	0.35
Fat mass (%)	NA	22.44 ± 1.81	26.88 ± 1.65	0.08

rs633265	G/G	G/T	T/T	*p* value
Age (years)	40.66 ± 3.09	37.15 ± 1.53	26.85 ± 1.0	0.01
BMI (kg/m^2^)	27.96 ± 1.25	29.43 ± 1.48	29.21 ± 4.48	0.67
Fat mass (%)	25.33 ± 1.60	25.71 ± 1.73	21.74 ± 6.33	0.38

rs1350341	A/A	A/G	G/G	*p* value
Age (years)	26.85 ± 1.0	37.15 ± 1.53	40.66 ± 3.09	0.01
BMI (kg/m^2^)	29.21 ± 4.48	29.43 ± 1.48	27.96 ± 1.25	0.67
Fat mass (%)	21.74 ± 6.33	25.71 ± 1.73	25.33 ± 1.60	0.38

**Table 6:** The genotypes distribution in the meal-test substudy population participated in the high-carbohydrate (HC) and control (C) meal tests

rs12970134	A/A	A/G	G/G
*N* (HC/C)	1/1	23/11	23/12
Genotype frequency (HC/C)	0.021/0.041	0.489/0.459	0.490/0.500

rs17782313	C/C	C/T	T/T
*N* (HC/C)	0/0	20/11	27/13
Genotype frequency (HC/C)	NA	0.426/0.458	0.574/0.542

rs633265	G/G	G/T	T/T
*N* (HC/C)	14/7	26/14	7/3
Genotype frequency (HC/C)	0.298/0.292	0.553/0.583	0.149/0.125

rs1350341	A/A	A/G	G/G
*N* (HC/C)	7/3	26/14	14/7
Genotype frequency (HC/C)	0.149/0.125	0.553/0.583	0.298/0.292

**Table 7:** The comparison of genotypes frequencies in general study cohort and meal test substudy population

rs12970134	rs1350341	rs17782313	rs633265
AA: 6.81e−01	AA: 6.77e−01	CC: 4.27e−01	GG: 1.00e + 00
AG: 5.11e−01	AG: 4.85e−01	CT: 6.33e−01	GT: 6.85e−01
GG: 7.66e−01	GG: 8.79e−01	TT: 1.00e + 00	TT: 5.89e−01

The carriers of genotype CC in rs17782313 presented the highest visceral fat accumulation (Table 1), and post-hoc analyses showed significant differences between CC and TT variants (*p* = 0.028). Between CC and CT carriers, as well as between CT and TT we noticed some trends (*p* = 0.059 and *p* = 0.101, respectively). The carriers of CC genotype showed also higher VAT/SAT ratio (Table 1). In post-hoc analyses we have noticed significant differences between CC and TT genotypes (*p* = 0.048), as well as significant differences between carriers of CT and TT variants (*p* = 0.013). Between CC and CT genotypes we did not find any significant differences (*p* = 0.211). We have not noticed any associations between rs17782313 and energy intake, dietary intake, physical activity, energy expenditure, nor substrate utilizations during meal challenge tests (data not shown).

The highest VAT content was observed also in carriers of TT genotype in rs633265 (Table 2). In the post-hoc analysis the significant differences were noted between GG and TT genotypes (*p* = 0.009), as well as between GT and TT variants (*p* = 0.023). Between GG and GT genotypes we did not find any differences (*p* = 0.526). The TT carriers presented also strong tendency to the highest VAT/SAT ratio (*p* = 0.05). We have not noticed any associations between rs633265 and energy intake, dietary intake, physical activity, energy expenditure, nor substrate utilizations during meal tests (data not shown).

Analysis dependently on rs12970134 genotypes showed that the homozygous carriers of allele A presented the highest BMI, total body fat mass, visceral and subcutaneous fat content, as well as VAT/SAT ratio (Table 3). The post-hoc analysis showed significant differences in BMI between AA and GG variants (*p* = 0.01), as well as between AA and AG genotypes carriers (p = 0.032). Between AG and GG variants differences were not significant (*p* = 0.32). The AA genotype carriers presented the highest total body fat content (Table 3), and significant differences were observed between AA and GG variants (p = 0.010), as well as between AA and AG (*p* = 0.012), but not between AG and GG genotypes carriers (*p* = 0.95). We have noted also the differences in body fat distribution and VAT content between AA and GG genotypes (*p* = 0.035). Between AA and AG genotypes the differences in post-hoc analysis were not so pronounced, but we have noticed some trends (*p* = 0.06). Between AG and GG variants we did not find any significant differences (*p* = 0.309). The carriers of AA genotype presented also the highest SAT content (Table 3). In the post-hoc analysis we noticed the differences between AA and GG genotypes (*p* = 0.002), as well as between AA and AG variants carriers (*p* = 0.0008). Between AG and GG variants we did not find any significant differences (*p* = 0.32). The AA genotype carriers presented also the highest VAT/SAT ratio (Table 3), however the post-hoc analysis showed significant differences only between AG and GG variants (*p* = 0.032). Between AA and GG genotypes carriers, as well as between AA and AG, we did not notice any significant differences (p = 0.18 and *p* = 0.47, respectively). We have not observed any associations between rs12970134 and energy intake, dietary intake, physical activity, energy expenditure, nor substrate utilizations during meal tests (data not shown).

**Fig. 1:**
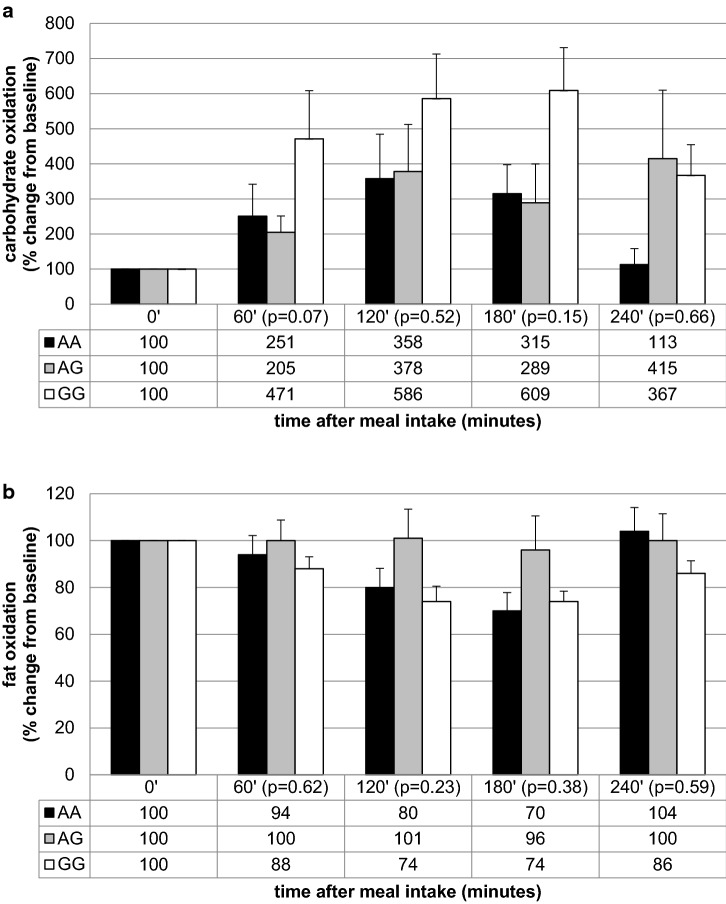
Association of MC4R rs1350341 genotypes with relative increase in carbohydrate (**a**) and fat (**b**) oxidation after HC meal

**Fig. 2:**
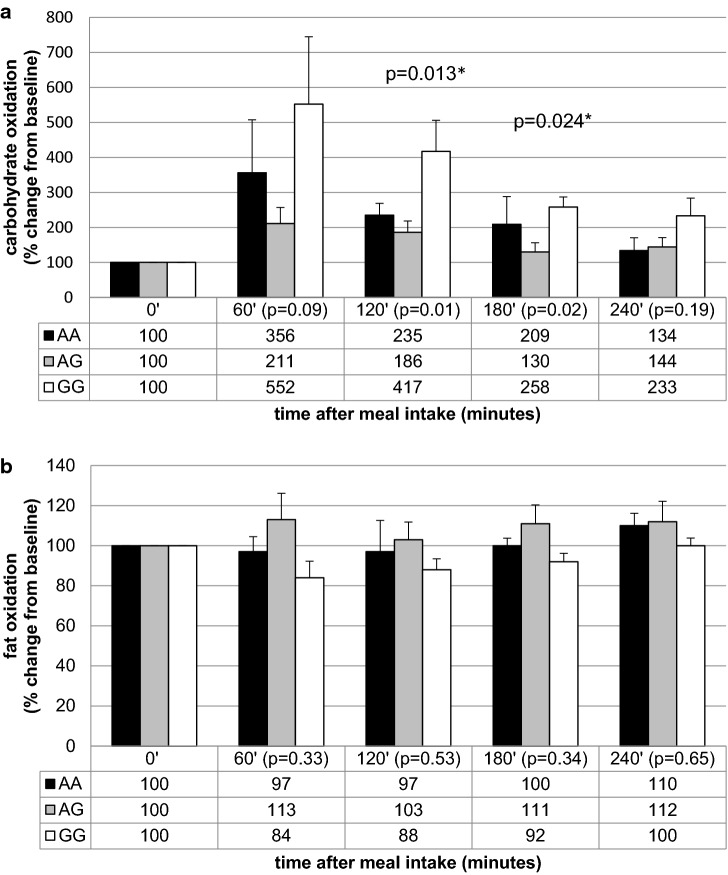
Association of MC4R rs1350341 genotypes with relative increase in carbohydrate (**a**) and fat (**b**) oxidation after C meal intake. *Post-hoc analysis for differences between GG and AG genotypes

Further analysis showed that the homozygous carriers of allele A in rs1350341 presented the highest VAT accumulation (Table 4) and the significant differences we have observed between carriers of AA and GG variants (*p* = 0.01), as well as between AA and AG genotypes (*p* = 0.03). Between AG and GG variants the differences were not significant (*p* = 0.5). The carriers of AA genotype presented also the highest VAT/SAT ratio (*p* = 0.046, Table 4), and the significant differences in VAT/SAT ratio were noted between AA and GG variants carriers (*p* = 0.02). Between AA and AG carriers, as well as between AG and GG we noticed just some trends (*p* = 0.17 and *p* = 0.13, respectively). We observed also a tendency for higher SAT accumulation in GG genotype carriers (*p* = 0.09, Table 4). The results from the meal challenge tests showed that he GG genotype carriers presented significantly lower fasting carbohydrate utilization and higher fasting fat oxidation (Table 8). When we analyzed the percentage change from the baseline, we found that homozygous men of G allele presented the higher relative increase in carbohydrate utilization after both meals intake (Figs. 1a, 2a). We did not observe any differences in the percentage change of postprandial fat oxidation (Figs. 1b, 2b). We have not noted any differences in energy expenditure at fasting state, nor in the postprandial change of energy expenditure after both meals intake (data not shown).

**Table 8:** The association between MC4R rs1350341 genotypes and fasting energy expenditure, carbohydrate and fat utilization, before high-carbohydrate (HC) and control (C) meals intake

rs1350341	A/A	A/G	G/G	*p* value
HC meal				
*N*	7	26	14	
Fasting energy expenditure (kcal/min/ffm)	0.019 ± 0.001	0.019 ± 0.001	0.018 ± 0.000	0.76
Fasting carbohydrate utilization (mg/min/ffm)	0.61 ± 0.13	0.97 ± 0.15	0.34 ± 0.06	0.01
Fasting fat utilization (mg/min/ffm)	1.34 ± 0.11	1.17 ± 0.08	1.35 ± 0.06	0.26
C meal				
*N*	3	14	7	
Fasting energy expenditure (kcal/min/ffm)	0.019 ± 0.001	0.018 ± 0.001	0.019 ± 0.000	0.59
Fasting carbohydrate utilization (mg/min/ffm)	0.57 ± 0.18	0.92 ± 0.15	0.40 ± 0.08	0.07
Fasting fat utilization (mg/min/ffm)	1.34 ± 0.06	1.10 ± 0.06	1.39 ± 0.07	0.01

For all of the investigated SNPs, in the meal-test substudy population, we did not notice any significant differences between carriers of different genotypes in fasting glucose concentrations, insulin levels, HOMA-IR, VAT, SAT contents, nor VAT/SAT ratios (data not shown), and we did not observe any differences in the calculated area under the curves (AUCs) of postprandial energy expenditure, carbohydrate and fat utilization levels (data not shown).

## Discussion

The MC4R is a key regulator of energy balance through functionally divergent central melanocortin neuronal pathways, influencing food intake and energy expenditure [19]. Our study showed that all of the investigated SNPs near the MC4R gene are associated with body fat distribution, and some of them with body fat content.

The higher visceral fat accumulation we have noticed in men carrying allele C in rs17782313, and the highest VAT was observed in the homozygous subjects. The allele C carriers presented also significantly higher VAT/SAT ratio, what means that more of the body fat is deposited viscerally. What is interesting, we have noticed associations between rs17782313 and body fat distribution despite the lack of any significant associations between this common SNP and BMI, WHR, as well as total body fat content. Qi et al. [24] and Zobel et al.[40] showed that rs17782313 near MC4R gene is associated with higher BMI, what was not observed in our study, maybe due to too small study sample. However, the higher waist circumference and abdominal obesity in carriers of C alleles, which were observed in the mentioned studies, might be associated with higher visceral fat accumulation, what has been confirmed by our results. The increase in visceral fat content is a major risk factor for type 2 diabetes mellitus, and Qi et al. [24] noted that the allele-C was associated with the 14% (2–32%) increased risk of T2DM. Moreover, the visceral fat accumulation, as well as diet, may lead to the insulin resistance (IR) development, and moreover, the authors found that CC genotype women presented higher energy, total fat (and percentage of energy from total fat) and protein intake [24]. The other authors [41] observed an association of the C-allele with a higher prevalence of snacking. Valladares et al. [42] found an association between rs17782313 genetic variants and the scores of enjoyment of food subscale, satiety responsiveness subscale, but without any differences in emotional over- nor under-eating, food fussiness, food responsiveness in children. Moreover, in a detailed examination authors noted that a very low satiety responsiveness scores, and very high scores for the enjoyment of food subscale accompanied the CC genotype, what may lead to over-eating. In our population we did not find any associations between rs17782313 genotypes and energy intake, dietary intake nor physical activity, and also study by Hasselbalch et al. [21] did not confirm those findings. It is worth to notice that in that study only women were included and, as it was observed, the sex differences may influence the associations between genetic variations and dietary intake. We have not noted any associations between rs17782313 and energy expenditure, nor substrate utilizations during meal tests, what is consistent with Kring et al. [28] study results, in which authors did not find any significant associations between energy expenditure and rs17782313.

The highest visceral fat content and strong tendency to the highest VAT/SAT ratio we have observed also in carriers of TT genotype in rs633265. What is also worth no notice, we have observed the differences in fat distribution without any significant differences in the percentage of total body fat content, neither in BMI nor WHR. Grant et al. [43] found that rs633265 was associated with childhood obesity in European American, what we did not observed in our adult European population, however we found that it influences the body fat distribution.

When we analyzed data dependently on genetic variants in rs12970134, we have noted that the homozygous carriers of allele A presented the highest BMI, total body fat mass, visceral and subcutaneous fat content, as well as VAT/SAT ratio. Our results are consistent with Zobel et al. [40] findings, which showed that the minor A-allele of rs12970134 was associated with overweight, obesity, morbid obesity, as well as abdominal obesity. We did not find any associations between genotypes of rs12970134 with dietary intake nor with physical activity (data not shown). Also Hasselbalch et al. [21] did not notice any associations between this genetic variants and total food intake, even if they found a positive association with intake of energy from the whole grain.

When we analyzed our results dependently on rs1350341 genotypes, we noted that the homozygous carriers of allele A presented the highest visceral fat accumulation, even if we did not find any differences in total body fat content, BMI nor WHR. The carriers of AA genotype presented also the highest VAT/SAT ratio. We have observed also tendency for higher SAT accumulation in GG genotype carriers, what may be a possible way of protection against visceral fat deposition [44]. The GG genotype carriers presented the lowest visceral fat content, and conversely, the homozygotes for the risk allele A, which presented the highest VAT accumulation and VAT/SAT ratio. The results obtained from the food diaries, and physical questionnaires were not statistically significant, and we observed just some tendencies, maybe because these data was collected from the questionnaires, and most probably to evaluate any potential associations and to minimize the possible estimating errors, the larger study sample would be needed, what is a major limitation of our study.

The analysis of results from the meal-test substudy showed that in fasting state the GG genotype carriers presented significantly lower carbohydrate utilization and higher fat oxidation. We analyzed the diet-induced thermogenesis and postprandial substrate utilization, but because we had noticed the different values at fasting state, therefore we decided to analyze the results as a percentage change from the baseline (the relative increase from the fasting state). We found that homozygous men of G allele presented higher relative increase in carbohydrate utilization after both meals. The homozygous A allele carriers were younger, but we have noted significantly differences or trends between AG and GG variants carriers, most probably due to too small numbers in AA genotype variant group, what is also a limitation of our study. A low allele frequencies in general population, and therefore in our study population, could be the limiting factors, and we must be cautious when extrapolating our results. We did not observe any differences in the percentage change of postprandial fat oxidation, nor in the fasting and postprandial energy expenditure. Observed in our experiment the highest relative increase in carbohydrate utilization after meal intake in GG genotype carriers (rs1350341), may protect against conversion glucose into the fat, as a result of de novo lipogenesis [45, 46], what was not noted in the AA genotype men. It can be an explanation why the carriers of AA genotype presented diminished ability to glucose utilization after meal intake, what could appear due to the higher VAT content and possible impaired insulin sensitivity, or on the other hand, it can also lead to exceed fat accumulation. Moreover, because we did not find any differences in the total body fat content, and we have noted some trends to the lower SAT content in AA genotype men, we can hypothesize that maybe the carriers of this SNP may have a lesser subcutaneous lipid storage capacity, what can be a reason of visceral fat accumulation and it’s clinical consequences, such as metabolic syndrome [47], or the higher risk of T2DM, what has been shown for carriers of this SNP in rs1350341 near the MC4R gene [24]. Some of the investigated SNPs are located close to the non-coding regions of DNA, and the potential associations between adiposity-related traits and non-coding variations are discussed [48–50]. The non-coding regions are very interesting and intriguing, but molecular mechanisms for these associations have yet to be clarified, with remembering that they can be in strong LD with causal variants of coding genes regions [48, 50]. The further genomic work is needed to experimentally determine the role of the non-coding DNA regions, taking into consideration also the gene–gene and gene-environment interactions.

## Conclusions

In conclusion, in the present study, we have examined the associations between the single-nucleotide polymorphisms near the MC4R gene and body fat content, body fat distribution, food intake, physical activity and metabolic differences in postprandial state. To our knowledge, the association between these common MC4R genetic variants and postprandial energy expenditure and substrate utilization has not been examined previously. We found the significant associations of investigated SNPs with body fat distribution, and we observed that investigated SNPs near MC4R gene may modulate postprandial metabolism. The GG (in rs1350341) genotype subjects, who presented the lowest visceral fat accumulation, presented also significantly higher relative increase in postprandial carbohydrate utilization, than AA genotype carriers. These observations deserve further investigations. We hope that our study may help to understand the pathways that control body fat deposition in humans, and to provide personalized treatment and prevention strategies to fight against obesity and its consequences, such as T2DM.
